# Supplementary material for: Model and Data Concur and Explain the Coexistence of Two Very Distinct Animal Behavioral Types
Source: Biology (Basel). 2020 Aug 21;9(9):241. doi: 10.3390/biology9090241 (PMC7564360; doi:10.3390/biology9090241)
Supplement: Supplementary file 1 [file biology-09-00241-s001.zip › biology-894806-supplementary/Ungoliant 25-07-20 SUPP MAT submitted.docx]

Sensitivity analysis

We ran a detailed series of simulations in order to disentangle which parameter values were more important in determining the strategy more likely to prevail. We focused on the two most competitive strategies of interest (EARLY-SPOV *vs*. MFCL), and the procedure was as follows. We interchanged one of the parameters that differed between poor and rich environments at a time (i.e. we assigned the value originally in rich environments to poor environments and *vice versa*) and reran all the simulations in the basic scheme for maintenance (one run of the basic scheme for each of the interchanged parameters: prey availability, “pspov”, “maxenc” and “mort”; see Table 1). This allowed us to reveal what parameters were more important at determining which strategy was maintained in either rich or poor environments. Furthermore, we took these parameters for rich and poor environments and when possible, ran the basic simulation scheme by either multiplying each of the parameters (one parameter at a time for each run) by 3, or dividing it by 3.

We also contrasted the pure SPOV strategy against MFCL when SPOV females were –rather unrealistically- 44% as large as MFCL females and when adult feeding did not affect MFCL reproductive output. This was to see if at an extreme in differences in body sizes and with fecundity only affected by fixed adult body size, as assumed by [20], the pure SPOV strategy could be maintained in natural populations.

Finally, because we found the parameter “maxenc” to be most responsible for which strategy persisted in poor or rich environments, we ran a last simulation in which we multiplied maxenc by 3 and started with pallele=0.01 for EARLY-SPOV against MFCL. This simulation was set to establish weather a very high probability of encounter between the sexes –which was found to be critical for EARLY-SPOV during sensitivity analysis- could widen the set of scenarios in which EARLY-SPOV can invade MFCL.

We also ran several other simulations with different parameter values, but since the results did not change our conclusions qualitatively, we do not mention the results here (see Table A1).

Table A1 (Supplementary Materials) summarizes the results of the sensitivity analysis, which revealed that the parameter most responsible for the differences found in which strategy was maintained in either rich or poor environments was “maxenc”, as we found that when this parameter was switched between environments, in one of the cases (“different” = 0.7) the opposite strategy was maintained (Table A1 column R). Thus, in our simulations within the main text, EARLY-SPOV prevailed in rich environments because the probability of encounter is high. Another parameter that is important for the prevalence of EARLY-SPOV in rich environments is “pspov”, as switching to lower probabilities of attack by EARLY-SPOV in a poor environment, this strategy was able to be maintained in one additional case (“different”=0.7 and recessive EARLY-SPOV, Table A1 column S). Prey availability also had an important effect for the prevalence of the MFCL strategy, as switching to high prey availability in an otherwise poor environment made MFCL to prevail in front of EARLY-SPOV (in the case for “different”=0.9 and recessive EARLY-SPOV, Table A1 column U). However, “maxenc” was a much important parameter, as when this parameter was high, higher prey availability was not enough in the basic scheme for MFCL to prevail when juvenile EARLY-SPOV survival was substantially high (“different” = 0.7, 0.9 and recessive EARLY-SPOV; compare Table A1 columns R and U). Furthermore, mutiplying “maxenc” by 3 resulted in EARLY-SPOV persisting in populations in 3 new combinations of parameters and also added one new frequency-dependent equilibrium and another quasi-equilibrium (Table A1 column Q). The higher mortality of EARLY-SPOV adult females (parameter “mort”) had no effect between environments, as switching this parameter did not change any qualitative result (Table A1 column T). However, multiplying the differential mortality of adult females (“mort”) by 3 (i.e., 0.0030*4.5 to determine the mortality rate of SPOV females), did result in two new scenarios in which MFCL females persisted in populations in front of EARLY-SPOV (“different”=0.9 in poor environments and “different”=0.5 in rich enviroments, Table A1 column W). Dividing “mort” by 3 did not provide qualitatively different results than the basic scheme of Fig. 2 (Table A1 column V). Multiplying prey availability by 3 also resulted in MFCL persisting in the same two additional scenarios as when increasing the relative mortality of EARLY-SPOV females (“different”=0.9 in poor environments and “different”=0.5 in rich environments, Table A1 column X). However, dividing prey by 3 had no qualitative effects (Table A1 column Y). Dividing the probability of EARLY-SPOV attack upon an approaching male (“pspov”) by 3 resulted in MFCL prevailing in two additional scenarios in front of EARLY-SPOV (“different”=0.5, 0.7 in rich environments and recessive EARLY-SPOV, Table A1 column AB).

When we simulated very large pure SPOV females, we found that in rich environments and with very high survival of juvenile SPOV (“different”=0.9), the SPOV strategy could be maintained either in a frequency-dependent equilibrium with MFCL (dominant SPOV) or even alone (recessive SPOV, Table A1 column M).

When the male-female encounter rate was multiplied by 3, we found that a mutation of dominant EARLY-SPOV arising in a pure population of MFCL (i.e., starting frequency of EARLY-SPOV allele of 0.01), could invade the population in two additional scenarios as compared with the scheme investigating the evolution of strategies in Fig. 4, one in which the MFCL strategy went extinct (“different”=0.9 in rich environments) and another in which both strategies were maintained in a frequency-dependent equilibrium (“different”=0.7 in rich environments, Table A1 column AK). Thus, encounter rates between males and females can also affect the evolution of strategies, even leading to coexistence (see also Table A1 column AJ for an unrealistic encounter rate).
